# The helicase and ATPase activities of RECQL4 are compromised by mutations reported in three human patients

**DOI:** 10.18632/aging.100506

**Published:** 2012-12-04

**Authors:** Martin Borch Jensen, Christopher A. Dunn, Guido Keijzers, Tomasz Kulikowicz, Lene Juel Rasmussen, Deborah L. Croteau, Vilhelm A. Bohr

**Affiliations:** ^1^ Center for Healthy Aging, Department of Cellular and Molecular Medicine, University of Copenhagen, 2200 Copenhagen, Denmark; ^2^ Laboratory of Molecular Gerontology, National Institute on Aging, Baltimore, MD 21224, USA

**Keywords:** RecQ helicase, RECQL4, Rothmund-Thomson Syndrome, RAPADILINO Syndrome

## Abstract

RECQL4 is one of five members of the human RecQ helicase family, and is implicated in three syndromes displaying accelerating aging, developmental abnormalities and a predisposition to cancer. In this study, we purified three variants of RECQL4 carrying previously reported patient mutations. These three mutant proteins were analyzed for the known biochemical activities of RECQL4: DNA binding, unwinding of duplex DNA, ATP hydrolysis and annealing of simplex DNA. Further, the mutant proteins were evaluated for stability and recruitment to sites of laser-induced DNA damage. One mutant was helicase-dead, had marginal ATPase activity and may be structurally compromised, while the other two showed greatly reduced helicase and ATPase activities. The remaining biochemical activities and ability to recruit to damage sites were not significantly impaired for any of the mutants. Our findings demonstrate a consistent pattern of functional deficiency and provide further support for a helicase-dependent cellular function of RECQL4 in addition to its Nterminus-dependent role in initiation of replication, a function that may underlie the phenotype of RECQL4-linked disease.

## INTRODUCTION

**Figure 1:**
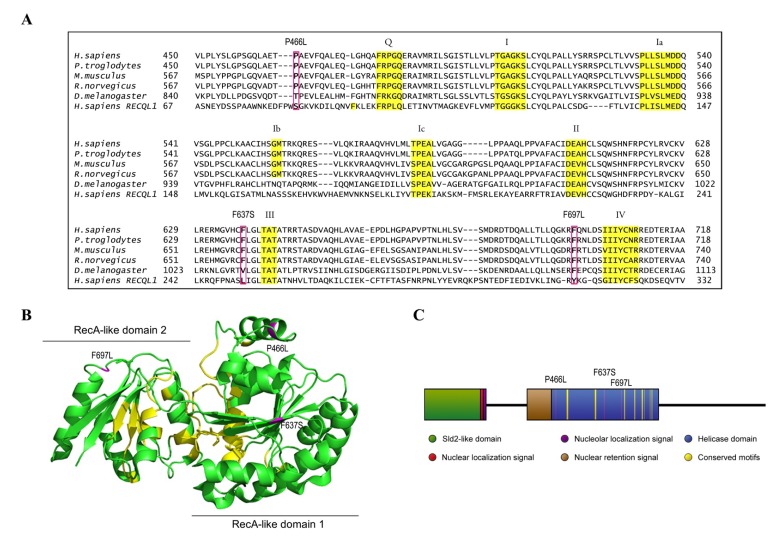
Homology map and model structure of RECQL4 (**A**) RECQL4 homologues across several species, illustrating the highly conserved nature of the amino acids examined in this study. Important helicase motifs are highlighted in yellow [50], while mutations are boxed in magenta. The sequence of RECQL1 is included to show the alignment used to create the model structure in **B**. (**B**) The crystal structure of RECQL1 without the RQC domain that is not present in RECQL4 (PDB ID: 2WWY), and with the amino acids homologous to the examined mutations in RECQL4 highlighted in magenta. Labels indicate the mutations in RECQL4. Important helicase motifs are highlighted in yellow, and the RecA-like domains 1 and 2 are indicated. (**C**) Domain map of RECQL4, with the examined mutations highlighted in magenta, and with important helicase motifs highlighted in yellow.

The RecQ family of helicases is conserved across multiple species and has been firmly linked to genomic maintenance [1–3]. Five RecQ helicases are present in humans: RECQL1, Bloom (BLM), Werner (WRN), RECQL4 and RECQL5. A central helicase domain, which allows for 3' to 5' unwinding of DNA, is conserved across the family [1,4]. In addition, RECQL4 possesses an N-terminal domain homologous to the *S. cerevisiae* protein Sld2 [5], the RecQ C-terminal (RQC) domains thought to affect protein interactions of the other members of the RecQ helicase family (Fig. 1C) [1,4,6,7]. Of the five human RecQ helicases, three (WRN, BLM and RECQL4) are associated with diseases involving segmental premature aging and cancer predisposition [8–11]. While the mechanisms behind Werner (OMIM 277700) and Bloom Syndromes (OMIM 210900) are not yet fully understood, the responsible proteins are at this stage relatively well-described as being required to resolve DNA secondary structures, and prevent inadvertent homologous recombination, respectively [12–19]. In contrast, the biological role of RECQL4 is not nearly as well understood [5,20–25].

Although RECQL4 has the same conserved helicase domain as the other RecQ helicases, *in vitro* experiments did not initially reveal any DNA helicase activity [24,26]. Weak ATP-dependent unwinding was eventually demonstrated, first in the presence of single-stranded competitor DNA [27] and later in the absence of the competitor [28,29]. Like the other RecQ helicases, RECQL4 exhibits strong strand annealing activity [26], which masks the helicase activity when longer duplex substrates are used. The extent to which the helicase activity of RECQL4 is critical for its biological role is not clear. A well-established role of RECQL4 is in the initiation of replication, where it interacts with the MCM10 protein and plays an essential part in assembling the CDC45-MCM2-7-GINS replication complex [20,30]. While the interaction with MCM10 was reported to inhibit the helicase activity of RECQL4 [20], mutants of*Drosophila* and *Xenopus* RECQL4 lacking functional helicase domain were unable to restore viability of knockout cells [5,21,27]. On the other hand, the helicase domain was not required to restore viability of knockout chicken DT40 cells [31], and reported human patients with mutations/deletions in the helicase domain obviously demonstrate viable replication [10,32].

RECQL4 has been implicated in several DNA repair pathways, either by being required for the repair of certain types of DNA damage [33-35], or through interaction with known DNA repair factors [22,25,36,37]. It has been suggested that the helicase domain plays an important role in this function [21,31,38], but a specific mechanism has not been identified. Mutations in RECQL4 occur in three human diseases, Rothmund-Thomson syndrome (RTS, OMIM 268400), RAPADILINO syndrome (OMIM 266280) and Baller-Gerold syndrome (BGS, OMIM 218600). These syndromes have partially overlapping phenotypes, with bone defects common to all three but for instance poikiloderma/sparse hair common to RTS and BGS, and osteosarcomas common to RTS and RAPADILINO [39-41]. One might speculate that such a variety of phenotypes arising from mutations in a single protein indicate multiple functions for RECQL4, and that the similarities to Werner and Bloom syndromes suggest dysfunctional genomic maintenance [1,8]. Patient mutations generally lie outside of the Sld2-like domain, which appears to be crucial for RECQL4's role in initiation of replication. Also, several of these mutations create premature stop codons that may prevent expression of the protein entirely [39,41]. While it is difficult to predict exactly how a replication-impaired phenotype might manifest, the survival of human patients with mutations in RECQL4 stands in contrast to studies on *Drosophila*, where deletion of the Sld2-like domain eliminated viability [21,38,42,43].

In the present study we examined three RECQL4 mutations previously reported in human patients [41]. Each mutation represents a single amino acid substitution in a highly conserved residue of the helicase domain of RECQL4. After expressing and purifying the mutant proteins we evaluated their thermal stability, recruitment to DNA double-strand breaks, ability to bind, anneal and unwind DNA, as well as hydrolyze ATP. From these analyses we uncovered a consistent pattern of functional deficiency, which may serve as an initial step in uncovering the cellular origin of RECQL4 disease phenotypes.

## RESULTS

### Overview of patient mutations

There are only twelve described patient mutations in *RECQL4* amenable to biochemical characterization, as the majority of mutations are either splicing errors, or frameshifts which introduce premature stop codons [41]. Further, most patients have compound heterozygous mutations, which inevitably but unfortunately hampers attempts to link molecular studies to patient phenotypes. We elected to study the three patient mutations P466L, F637S, and F697L where the affected amino acids lie within the highly conserved helicase domain of RECQL4. These are highlighted in Fig. 1A, which also shows the conserved helicase motifs in yellow. The crystal structure of RECQL4 is not yet available, but to get an impression of where the affected residues are located we threaded the aligned sequence of RECQL4 onto the crystal structure of human RECQL1 (PDB ID: 2WWY), omitting the RQC domain, which is not present in RECQL4. Human RECQL4 shares 41% identity and 56% similarity with the helicase domain of human RECQL1 and the model structure is shown in Fig. 1B. Because the mutations lie so close to highly conserved motifs (Fig. 1), we expect their structural position in this map to resemble that of RECQL4, though we cannot say for certain which differences exist in the structures of RECQL1 and RECQL4. The three mutants (P466L, F637S and F697L) were successfully purified (Supp. Fig. 1), and are highlighted in magenta in Fig. 1.

### Overall structure of mutants appears to be conserved

In order to evaluate the structural stability of our mutants compared to WT, we analyzed their unfolding as a function of temperature by measuring the fluorescent signal of a SYPRO^®^ Orange protein binding dye. In this assay, melting curves can be extracted from an increase in fluorescent signal, which is observed as the protein unfolds to reveal additional dye binding sites [44] (see Supp. Fig. 2 for melting curves and raw data). While F637S showed clean single-step unfolding, both WT and the remaining mutants appeared to demonstrate more complex unfolding. The higher initial fluo-rescence observed from F637S may indicate partial structural destabilization by this mutation. While we were not able to accurately describe the unfolding of WT and the other mutants based on our data, it appears consistent with two-step unfolding. If that is the case, P466L may show an effect on the second unfolding step, though as mentioned our data cannot establish this conclusively. Nonetheless we estimated apparent melting points from all the melting curves by approximating single-step unfolding, and these results are summarized in Supp. Table 2. By this analysis, we did not see significant differences in melting temperature for any of the mutants as compared to the WT.

### All mutants are still able to bind DNA

**Figure 2:**
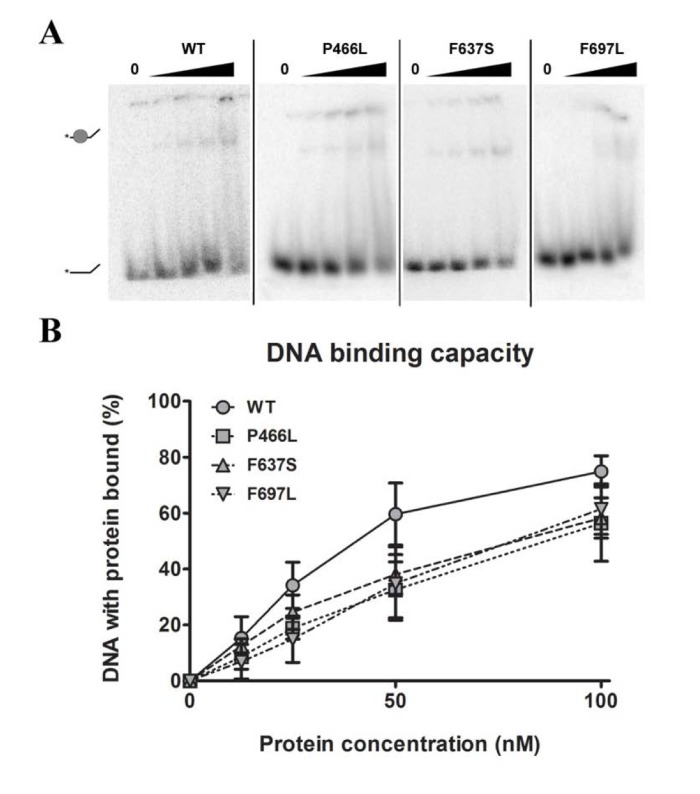
DNA binding is not significantly affected by mutations (**A**) Amalgamated gel from representative experiments showing DNA binding of WT and mutants at 0, 12.5, 25, 50 and 100 nM protein. (**B**) Binding data compiled from triplicate experiments. Mutants universally appear to have slightly reduced binding compared to wild-type, but the difference is not significant. Error bars represent standard error of mean from three experiments.

Being prerequisite for other types of activity, our first object of investigation was to test the ability to bind DNA. We chose to use single-stranded DNA as the most realistic model for RECQL4's DNA binding *in vivo*, and proceeded to test the binding of each mutant to a single-stranded 37-mer oligonucleotide at a range of protein concentrations. Fig. 2A shows a decrease in intensity of the lower band (free DNA), signifying protein binding and retardation of the DNA in the gel, leading to the appearance of a corresponding upper band representing protein-bound DNA. The upper band may smear considerably when the DNA-protein complex dissociates in the gel, and we therefore used the intensity of the lower band to quantify the level of unbound DNA. Although all three mutants showed a trend of slightly decreased binding (approximately 80% of WT at 100 nM protein, Fig. 2B), this difference was not statistically significant. We thus conclude that the mutants all show proficient DNA binding.

### Each mutation adversely affects helicase activity

**Figure 3:**
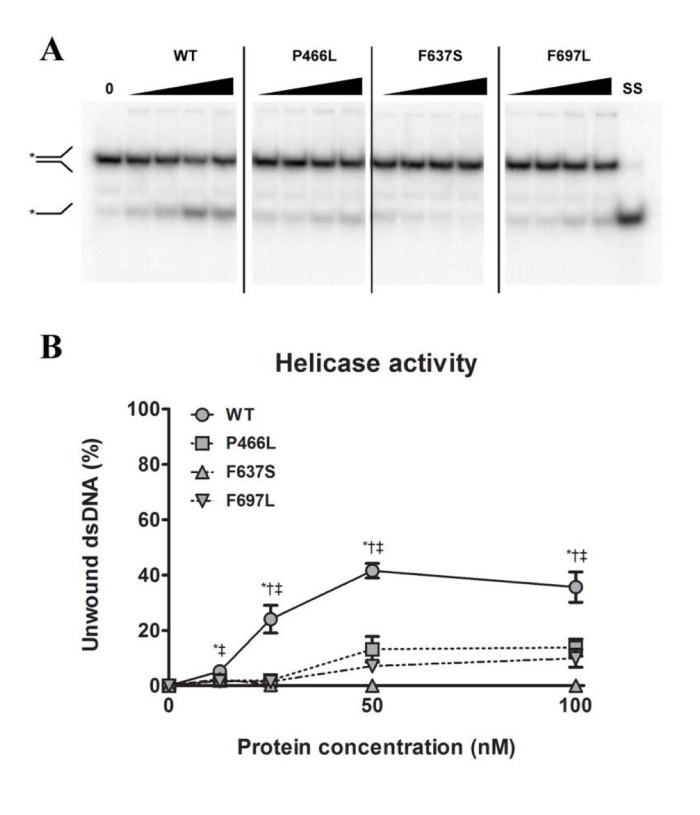
All mutants have decreased helicase activity (**A**) Amalgamated gel from representative experiments showing helicase activity of WT and mutants at 0, 12.5, 25, 50 and 100 nM protein, as well as a single-stranded control. (**B**) Unwinding data compiled from triplicate experiments. The P466L and F697L mutants show significantly reduced helicase activity compared to wild-type (38% and 28% of WT respectively at 100 nM protein), while F637S has no detectable activity. *, †, and ‡ denote p < 0.05 between WT and P466L, F637S and F697L, respectively. Error bars represent standard error of mean from three experiments.

Since substrate binding is a prerequisite for the 3'-5' DNA helicase activity of RECQL4, we next examined whether the mutant proteins were able to unwind a short DNA fork substrate. The substrate has a 22 bp duplex region followed by a 15 bp non-complementary region, and has previously been used to demonstrate helicase activity of RECQL4 in the absence of single-stranded competitor DNA [28]. For gels like those shown in Fig. 3A we calculated the relative intensity of the bottom (single-stranded) band versus the top (double-stranded) band, and plotted the results graphically in Fig. 3B. In contrast to the binding data, all three mutants showed significantly reduced ability to unwind this substrate, as compared to WT. For P466L and F697L we observed negligible activity below 50 nM protein, and activities ranging from 20 to 37% of WT at 50 and 100 nM protein. Meanwhile, F637S showed no detectable helicase activity. To confirm that the difference in activity was not an artifact of the purification we repeated the experiments using independent protein preparations based on an alternate purification protocol (described in the supplementary information), and observed the same pattern of activities (data not shown).

### Deficient helicase activity correlates with inability to hydrolyze ATP

**Figure 4:**
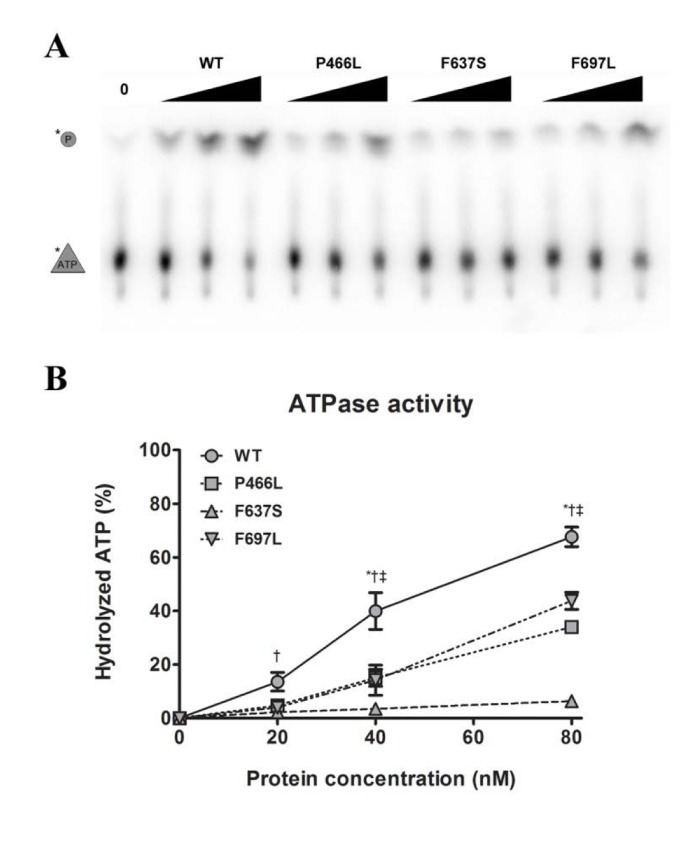
Decreased helicase activity correlates with lower ATPase activity (**A**) Representative gel showing ATPase activity of WT and mutants, at 0, 20, 40, and 80 nM protein. (**B**) ATPase data compiled from triplicate experiments. The activities of P466L, F697L and in particular F637S are significantly reduced compared to wild-type (at 50%, 65% and 9% of WT at 80 nM protein, respectively). *, †, and ‡ denote p < 0.05 between WT and P466L, F637S and F697L, respectively. Error bars represent standard error of mean from three experiments.

The helicase activity of RECQL4 is dependent on hydrolysis of ATP [26], which in turn requires the presence of DNA. Our next step was therefore to examine the ability of the mutants to hydrolyze ATP in the presence of DNA. We observed the cleavage of labeled phosphate (top band) from ATP (bottom band) during incubation with a fixed concentration of DNA and varying concentrations of protein (Fig. 4A). Plotting the data revealed a pattern similar to that observed for helicase activity. P466L and F697L showed significantly reduced activity as compared to WT, with ~40% of WT activity at 20 and 40 nM and peaking at ~50 and 65% at 80 nM protein for P466L and F697L, respectively (Fig. 4B). As we saw for helicase activity, the F637S mutant was even more strongly affected, peaking at approximately 10% of WT activity for 80 nM protein. This pattern was confirmed using the alternate protein preparation, though F637S was not quite as severely impacted.

### Strand annealing activity is not correspondingly affected by mutations

**Figure 5:**
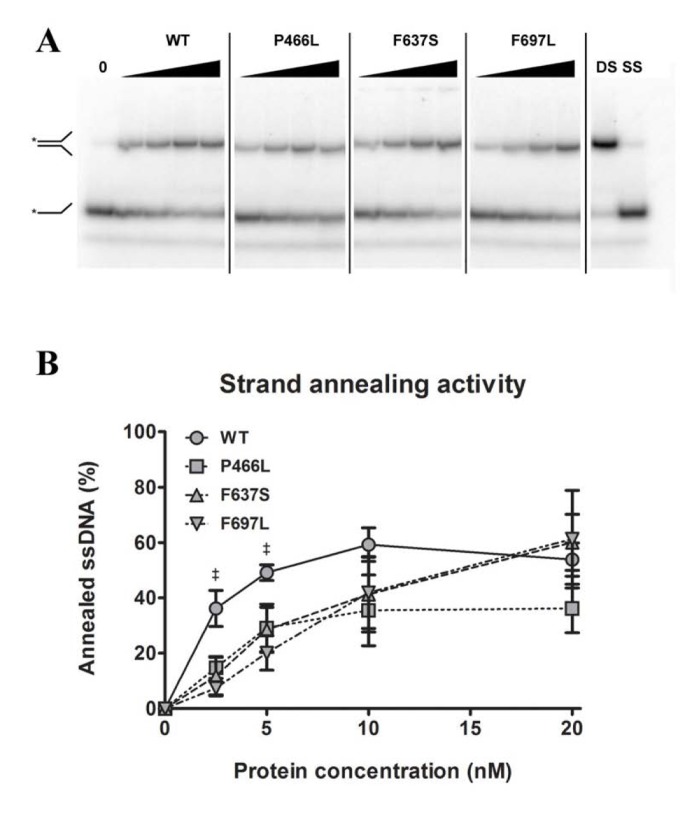
Strand annealing activity is not correspondingly reduced Amalgamated gel from representative experiments showing annealing activity of WT and mutants at 0, 2.5, 5, 10 and 20 nM protein, as well as double- and single-stranded controls. (**B**) Annealing data compiled from triplicate experiments. At 2.5 and 5 nM protein, WT shows significantly higher activity than F637S, but at higher concentrations this difference is not seen. ‡ denote *p* < 0.05 between WT and F697L. Error bars represent standard error of mean from three experiments.

Like the other RecQ helicases, RECQL4 can pair single-stranded DNA to double-stranded DNA. This annealing activity is in direct opposition to the DNA unwinding (helicase) activity, such that a variation in annealing activity could change the amount of unwound substrate in helicase assays and thereby lead to an apparent difference in helicase activity. To test this, we performed strand annealing assays utilizing a 5'-labeled single-stranded DNA substrate and a complimentary strand in the absence of ATP. We observed the reduction of the lower band (single-stranded DNA) and the corresponding appearance of the higher band (double-stranded DNA) with increasing protein concentration. Note that since the annealing activity of RECQL4 is stronger than the helicase activity, a much lower range of protein concentrations was used here. As shown in Fig. 5B, F697L showed significantly lower activity than WT, at 20 and 40% for 2.5 and 5 nM protein, respectively, while the other two mutants show an apparent but not significant decrease. At higher concentrations, corresponding to the lowest concentra-tions used in the helicase assays, there is no significant difference between WT and mutants (also confirmed with the alternate protein preparation).

### All mutants recruit to DNA double-strand breaks with equivalent dynamics

**Figure 6:**
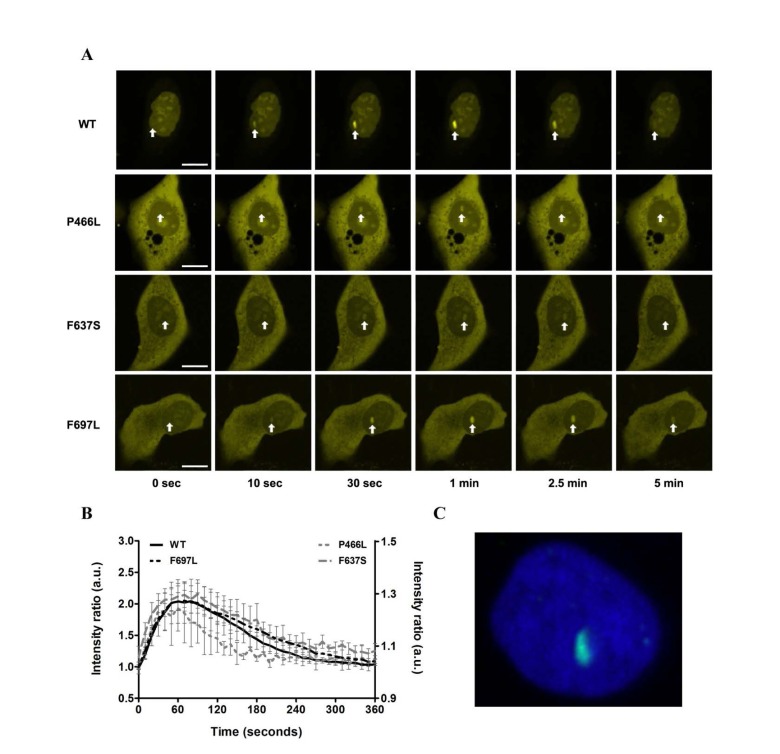
Recruitment to DNA double-strand breaks is not affected (**A**) The recruitment of either WT or mutant RECQL4 to DNA double-strand breaks induced by microirradiation is shown for six time points (0, 10, 30, 60, 150 and 300 seconds). Damage sites are indicated by white arrows. In all cases recruitment occurs rapidly, peaks after about one minute and has largely abated after five minutes. Scale bars are 10 μm. (**B**) Protein recruitment quantified as the ratio of intensities at the damage site compared to the rest of the nucleus. The absolute signal from five examined cells is plotted with standard error of mean. While there is variation in absolute signal, the recruitment and retention dynamics of WT and mutants are remarkably similar. Only P466L shows a trend of accelerated release from the damage site. WT and F697L are plotted on the left y-axis, P466L and F637S on the right. (**C**) Immunohistochemical staining of γH2AX (green) following laser irradiation of the nucleus (DAPI, blue) to confirm the creation of double-strand breaks.

To evaluate whether the mutant proteins are stable in human cells we expressed YFP-RECQL4 fusion proteins (WT and mutants) in U2OS cells, and as a functional test monitored recruitment to sites of double-strand breaks induced by micropoint laser irradiation [25]. We observed recruitment of both WT and mutant RECQL4 to the damaged site within ten seconds (Fig. 6A). Fig. 6B shows the time course of recruitment, with recruitment level represented as the ratio of signal at the damaged site vs. signal level of the rest of the nucleus. For both WT and mutants, accumulation saturated after about one minute and had mostly faded after five minutes. While there is variation in the absolute signal, the recruitment and retention dynamics of WT and mutants are very similar. Only P466L shows a trend of accelerated release from the damage site.

Trafficking of RECQL4 between cytoplasm and nucleus has been reported previously [45], and we observed varying relative distribution of RECQL4 in these two compartments. Although we did not examine cellular localization in detail, the WT protein showed predominantly nuclear localization more frequently than the mutants. Since the examined mutations do not fall within nuclear localization or retention signals (Fig. 1**C**) we speculate that a potential difference in localization might arise from the presence of a greater amount of non-fusion GFP in the cytoplasm, perhaps originating from partially degraded fusion protein (Supp. Fig. 3). Simply, we conclude that the mutant proteins localize to DNA damage sites with largely unaltered dynamics, albeit with the caveat that these mutants may show a different nuclear-cytoplasm distribution than WT RECQL4.

## DISCUSSION

Our data shows that the helicase and ATPase activities are strongly affected for all three mutants, similar to what was recently found for the c.1390+2delT mutation found in other RAPADILINO patients that causes a 44 amino acid deletion just prior to the helicase domain [46]. In view of the ATP-dependent nature of the helicase activity, this could be interpreted in two ways: either a given mutation prevents ATP hydrolysis, which in turn inactivates the helicase function, or the mutation disrupts helicase activity, which results in ATP not being hydrolyzed regardless of the capacity for doing so. Given that ATPase activity was measured in the presence of single-stranded DNA that would not provoke unwinding, we find the first option most likely. While our sample size is limited, it is noteworthy that all three mutations seem to target the same enzymatic capacity; it might be edifying to examine additional patient mutations in this manner to see how broadly the pattern fits.

It must be noted that at low protein concentrations WT showed higher strand annealing activity than the mutants. While this may be of consequence on its own, it is unlikely that it would influence our observations regarding helicase activity: the difference in annealing activity was evident only at protein concentrations low enough that effectively no helicase activity was observed, while at concentrations comparable to those used in the helicase assay the annealing activity of WT and mutants had converged. From our data we cannot say whether annealing activity is in itself important for the biological role of RECQL4, nor whether this function is affected in some human patients.

Although the examined mutants display very similar patterns of activity, minor differences do emerge in the various assays. These presumably arise from the differences in position and physiochemical properties of the mutated residues. Based on the model structure of shown in Fig. 1B, we can speculate on the observed changes in activity and the location of each mutation examined in this study. In the model structure, P466 is located at the N-terminus of an α-helix, relatively close to the ATP binding region. Proline residues at α-helix N-termini are known to stabilize proteins [47], and are also well known helix-breakers. Therefore, the P466L substitution may produce an aberrant elongation of the α-helix which could disrupt local structure enough to impair activity. Alternatively, the P466L substitution may loosen the α-helix, and this decrease in local rigidity may affect the ability to hydrolyze ATP. F637S occurs within a β-sheet that is fully buried in the helicase core (RecA-like domain 1). Thus, the substitution from aromatic to hydrophilic residue may destabilize the helicase core, and thereby affect the catalytic activity (indeed, F637S exhibited the least helicase and ATPase activity). F697 is located in a loop between an α-helix and a β-sheet in conserved motif IV (Fig. 1A). Since this β-sheet also forms the core of the RecA-like domain 2, the elimination of an aromatic side chain may again alter the local conformation of this region. These hypotheses are consistent with the structural data presented in Supp. Fig. 2 and Supp. Table 2, which suggests the possibility that F637S may be partially destabilized, while P466L and F697L appear more intact structurally.

In addition to the implicit relevance of human disease-associated mutations, the syndromes involving RecQ helicases are of interest because they display segmental premature aging and can arguably be used to study normal human aging. This is most obvious in Werner syndrome, where most mutations lead to truncation of the protein; Bloom syndrome is also most commonly the result of truncation, although loss-of-function missense mutations have been reported [7]. While some mutations in *RECQL4* also lead to loss of protein, both missense mutations and the deletion of exon 7 common in RAPADILINO syndrome [41] stand in contrast to the truncations of WRN and BLM. It is therefore noteworthy that the missense mutations examined here result in loss of function, in the same vein as mutations of WRN and BLM.

With this in mind, what can our results impart about the origin of disease in these patients? Two lines of reasoning present themselves. It could be argued that despite the relatively weak helicase activity displayed by RECQL4, this activity is crucial for the proper functioning of the protein. Because RECQL4's function in replication depends on the N-terminal region, and not on the helicase domain [31,42], this interpretation presupposes that RECQL4 serves more than one role in the cell, and that the observed phenotype stems from dysfunction in a role other than initiation of replication. This hypothesis is strongly supported by a recent study on cells expressing RECQL4 without the helicase and C-terminal domains; these cells replicate normally when unstressed, but are sensitive to ionizing radiation, which induces S-phase arrest [48]. This would suggest that at least the active helicase function is not required for RECQL4's role in replication, consistent with earlier observations [31]. It also does not rule out the possibility that RECQL4 could function as a passive helicase alongside MCM2-7, as has previously been suggested [21]. One argument to support this interpretation is that RECQL4 retains the (functional) helicase domain that defines the family; if this domain did not serve any cellular function, one would not expect it to have been evolutionarily conserved. Further, the fact that these mutations did not significantly reduce the capacity to bind DNA substrates could mean that the mutants bind their normal cellular substrate, but are unable to process it and thereby block the relevant pathway. This is particularly plausible in light of the opposed enzymatic activities for unwinding and annealing of DNA. Since the mutations did not reduce the annealing activity as they did the helicase, the mutant proteins might actively counteract the unwinding of a substrate.

Another interpretation is that while the helicase activity is affected in these mutants, this is a consequence of the deficient ATPase activity, and the helicase activity not important in itself. Rather, the ability to hydrolyze ATP is required for an independent function of the protein, and its deficiency produces the observed phenotype. This function could involve interactions with other proteins, possibly in a recruitment role. While it is impossible to draw extensive conclusions regarding this hypothesis based on the data presented here, the fact remains that only about two-thirds of patients with Rothmund-Thomson syndrome have mutations in *RECQL4*. Given that the disease phenotype can arise independently of *RECQL4* mutations, it should be reconciled whether the affected function of RECQL4 depends on another protein that is also impaired by a mutation causing the remaining third of RTS cases. Identifying interaction partners of RECQL4 could help to further test this hypothesis.

In conclusion, our findings reveal that mutations of RECQL4 from three RAPADILINO patients all reduce the helicase and the ATPase activities of the protein. These observations are consistent with the hypothesis that RECQL4 has one or more cellular functions in addition to its role in initiation of replication, functions which are dependent on the a functional helicase domain. Further study of RECQL4 patient mutations could resolve whether such a secondary function underlies the disease phenotypes.

## METHODS

### Protein expression and purification

The wild-type RECQL4 (WT) and the three mutants (P466L, F637S, and F697L) were expressed and purified identically. WT RECQL4 with a cleavable N-terminal glutathione S-transferase (GST) tag and a C-terminal 9-histidine tag was inserted into the pGEX6p1 vector (GE Healthcare), and mutants were generated by site-directed mutagenesis using PCR amplification, as described previously [28]. Plasmids were transformed into *E. coli* Rosetta2 (DE3) (Novagen), and cultures grown at 37°C until the OD_600_ reached 0.6. Protein production was induced by adding 0.3 mM isopropyl β-D-1-thiogalactopyranoside (IPTG), and cultures were further incubated at 16°C for 16 hours. The transformants were then harvested by centrifugation and stored at −80°C.

Purification was carried out as described previously [28], with the following modifications: the first column used was 140 mL MonoQ to accommodate a larger volume of lysate, and elution from the SP sepharose column was done with a 250-500 mM KCl gradient. Protein concentrations were determined by gel analysis combined with bicinchoninic acid (BCA) assays.

An alternate protocol for expression and purification was used to verify that any difference in activity within independent protein preparations was not an artifact of the purification. The alternate protocol is described in the supplementary information. We attempted purification of three additional RECQL4 mutants (R522C, R522H and L678P), but these turned out to be unstable using both the original and alternate purification protocols (see Supp. Fig. 1) and were not subjected to further analysis.

### Oligonucleotide substrates

Oligonucleotides were synthesized and PAGE purified by Integrated DNA Technologies (Coralville, IA), with sequences listed in Supp. Table 1. Indicated strands were 5' radiolabeled with [γ-^32^P] ATP (PerkinElmer Life Sciences) using T4 polynucleotide kinase (New England Biolabs). Unincorporated [γ-^32^P] ATP was removed using MicroSpin G-25 columns (GE Healthcare). To create fork and full duplex substrates, corresponding oligo-nucleotides were combined in annealing buffer (50 mM Tris-HCl pH 7.0 and 25 mM KCl) in a 1:2 ratio of labeled to unlabeled oligonucleotide, heated to 90 °C for 10 minutes and cooled slowly to room temperature.

### Enzymatic assays

For DNA binding assays, RECQL4 (amount indicated in figure legends) was incubated in reaction buffer (25 mM Tris HCl, pH 7.4, 50 mM KCl, 0.1 mg/mL bovine serum albumin (BSA), 1 mM DTT) with 0.5 nM labeled Fork-Top DNA for 15 minutes at room temperature. Reactions received glycerol to 17% for loading and were run at 4°C, 100V for 80 minutes on a 5% 29:1 acrylamide:bis-acrylamide gel in 0.5x Tris/Borate/EDTA buffer.

For helicase assays, RECQL4 (amount indicated in figure legends) was incubated in reaction buffer (30 mM Tris HCl, pH 7.4, 50 mM KCl, 5 mM MgCl_2_, 11% glycerol, 0.1 mg/mL bovine serum albumin, 1 mM DTT, 5 mM adenosine triphosphate (ATP)) with 0.5 nM labeled Fork-Top/Fork-Bottom for 30 minutes at 37°C. Stop dye (10 mM Tris HCl, pH 8.0, 10 mM EDTA, 10% glycerol, 0.3% sodium dodecyl sulphate (SDS)) was added to reactions before running at 125V for 120 minutes on a 12% 19:1 acrylamide:bis-acrylamide native gel in 1x Tris/Borate/EDTA buffer.

For ATPase assays, RECQL4 (amount indicated in figure legends) was incubated in reaction buffer (30 mM Tris HCl, pH 7.4, 50 mM KCl, 5 mM MgCl_2_, 11% glycerol, 0.1 mg/mL bovine serum albumin, 1 mM DTT, 50 μM (cold) ATP, 10 μM 10 μCi/μL [γ-^32^P] ATP) with 0.2 μM M13mp18 single stranded DNA (New England Biolabs) for 1 hour at 37°C. 167 mM EDTA was added to stop reactions before separating samples by thin-layer chromatography on Baker-flex Cellulose PEI sheets (J.T. Baker) for 45 minutes in 0.8 M LiCl, 1 M formic acid.

For strand annealing assays, RECQL4 (amount indicated in figure legends) was incubated in reaction buffer (30 mM Tris HCl, pH 7.4, 50 mM KCl, 5 mM MgCl_2_, 11% glycerol, 0.1 mg/mL bovine serum albumin, 1 mM DTT) with 0.5 nM labeled Fork-Top oligo and 0.5 nM unlabeled Fork-Bottom for 20 minutes at 37°C temperature. Stop dye (10 mM Tris HCl, pH 8.0, 10 mM EDTA, 10% glycerol, 0.3% SDS) was added to reactions before running at 125V for 120 minutes on a 16% acrylamide native gel in1x Tris/Borate/EDTA buffer.

All gels were exposed on storage phosphor screens (GE Healthcare) and scanned using a Typhoon 9400 imager (GE Healthcare). Resulting images were analyzed using ImageQuant 5.2 (GE Healthcare). All assays were performed in triplicate, and error bars plotted as standard error of mean. All graphs are normalized to the negative control as zero activity.

### Thermostability testing

Evaluation of the thermostability of WT and mutants was performed by measuring the increase in signal from SYPRO^®^ Orange protein stain (Life Technologies) upon temperature-induced protein unfolding [44]. 2 μg protein in 20 μl buffer containing 20 mM KH?_2_PO_4_ pH 7.5, 178 mM KCl, 6% glycerol, 1 mM DTT and 5x SYPRO® Orange were incubated in a MyIQ quantitative polymerase chain reaction (PCR) machine (BioRad), and subjected to 141 steps of 0.5°C increase in temperature every 15 seconds, for a total range of 20-90°C. Fluorescent signal was measured throughout, with peaks describing unfolding of the protein and the resulting increase in SYPRO® Orange binding. Melting curves were calculated from these peaks as described previously [44].

### Preparation of plasmids for fluorescence microscopy

Vector YFPc2 (Clontech) was digested with EcoRI and SalI, followed by insertion of either WT or a mutant gene (P466L, F637S or F697L) in an EcoRI and XhoI fragment from the pGEX6p1 vector described in section 2.1. The resulting vectors were verified by sequencing, and used to express YFP-RECQL4 (WT or mutant) fusion protein for microscopy, as described in section 2.6.

### MicroPoint laser irradiation and microscopy

U2OS cells were maintained in DMEM (Gibco), supplemented with 10% fetal bovine serum, penicillin (50 U/ml) and streptomycin (50 g/ml) (Gibco, Life technologies), and grown at 37°C in a humidified atmosphere containing 5% CO_2_. One day prior to transfection approximately 10^5^ cells were seeded in 15 mm dishes with thin glass bottoms (Mat-Tek). Cells were transfected with Lipofectamine LTX (Life Technologies Inc.) according to manufacturer's instructions using 1 μg of the relevant vectors. Targeted DNA damage was introduced using the MicroPoint^®^ Ablation Laser System from Photonic Instruments at 14% laser power (3.3 μW), and fluorescent protein recruitment and retention monitored as described previously [49]. Images were acquired every 10 seconds for at least 5 minutes. Immuno-histochemical staining to confirm the presence of double-strand breaks was performed as described previously [49].

## SUPPLEMENTAL DATA, TABLES AND FIGURES



## Abbreviations

BGS: Baller-Gerold syndrome
BLM: Bloom syndrome protein
HRDC: Helicase/RNAse D C-terminal
RQC: RecQ C-terminal
RTS: Rothmund-Thomson syndrome
WRN: Werner syndrome ATP-dependent helicase
