# Supplementary material for: The helicase and ATPase activities of RECQL4 are compromised by mutations reported in three human patients
Source: Aging (Albany NY). 2012 Dec 4;4(11):790–802. doi: 10.18632/aging.100506 (PMC3560432; doi:10.18632/aging.100506)
Supplement: Supplementary file 1 [file aging-04-790-s001.pdf]

SUPPLEMENTAL DATA

Alternate protein expression and purification

Human RecQL4 with N-terminal GST and C-terminal 9-His tags was expressed using the pGEX6p1 vector (GE Healthcare) in the E. coli strain Rosetta2 (DE3) (Novagen) as described in section 2.1. Frozen cell pellet from 8 L culture (~30 g) was resuspended in 150 mL of lysis buffer (50 mM HEPES, pH 7.5, 300 mM NaCl, 10% glycerol, 25 mM Imidazole, 1 mM PMSF, 1 tablet/50 ml Complete EDTA-free Protease Inhibitor Cocktail (Roche)), disrupted by sonication, spun 30 min. at 37,000g, and filtered through 45 µm membrane. All purification steps were carried out at 4°C using ÄKTApurifier instrument (GE Healthcare). Clarified lysate was loaded onto HisTrap FF 5 mL column equilibrated in Buffer A (50 mM HEPES, pH 7.5, 300 mM NaCl, 10% glycerol) supplemented with 25 mM Imidazole. The column was washed with 20 column volumes (CV) of Buffer A + 50 mM Imidazole, and the protein eluted with 10 CV of Buffer A + 250 mM Imidazole. Fractions containing RecQL4 were pooled, and loaded onto GSTrap 4B 5 mL column equilibrated in Buffer A. The sample was recirculated through the column for 1 hour to maximize binding. Next, the column was washed with 10 CV of Buffer A and developed with Buffer A supplemented with 50 mM reduced Glutathione. Peak fractions with RecQL4 protein were pooled, adjusted with DTT to 1 mM final concentration, and subjected to cleavage with 120 units of PreScission protease (GE Healthcare) for 15 hrs. at 4°C. Cleaved protein was diluted 1:6 (v/v) with Buffer A + 25 mM Imidazole and loaded onto HisTrap FF 5 mL column equilibrated in the same buffer. The column was washed with 30 CV of Buffer A + 75 mM Imidazole and eluted with 10 CV of Buffer A + 250 mM Imidazole. Fractions with purified RecQL4 protein were pooled and concentrated using Amicon® Ultra-4 centrifugal filter unit with 50 kDa cutoff (Millipore). Concentrated protein was diluted 1:1 with cold 100% glycerol, aliquoted and stored at -80 °C.

REFERENCE

1. Rossi ML, Ghosh AK, Kulikowicz T, Croteau DL, Bohr VA. Conserved helicase domain of human RecQ4 is required for strand annealing-independent DNA unwinding. DNA repair. 2010; 9:796–804.

Supplementary Table 2. Estimated melting points of WT and mutants. The melting temperatures of WT and mutants from the data in Supp. Fig. 2 using approximated single-step unfolding. Except for F637S no significant differences are apparent, suggesting that the mutant proteins retain the same overall structure. The change in Tm (ΔTm^{obs}) was calculated as follows: $\Delta Tm^{obs} = Tm^{obs}$ (mutant) $- Tm^{obs}$ (wild type). Experimental error, ± 2.5 °C, n=2.

Protein	T_m^{obs} (˚C)	ΔT_m^{obs} (˚C)
WT RECQL4	45.8	-
P466L	47.0	+1.2
F637S	49.3	+3.5
F697L	44.5	-1.3

$\Delta T_m^{obs} = T_m^{obs}$ (mutant) - T_m^{obs} (wild type)
Experimental error = ±2.5 ˚C, n = 2

Supplementary Table 1. Oligonucleotide

Oligo	Sequence (5'-3')
Fork-Top	GTAGTGCATGTACACCACACTCTTTTTTTTTTTTTTT
Fork-Bottom	TTTTTTTTTTTTTTTGAGTGTGGTGTACATGCACTAC

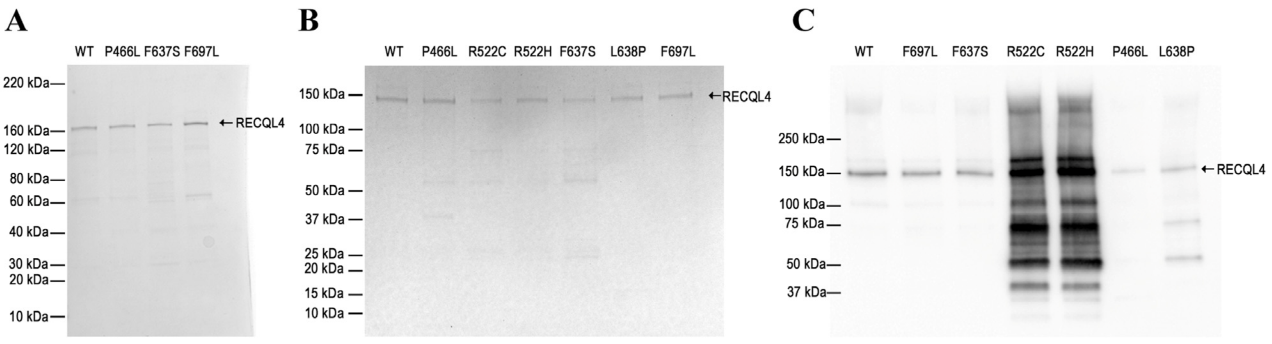

Supplementary Figure 1. Expression and purity of proteins. (**A**) Protein gel from the primary purification used for biochemical assays, showing WT RECQL4 and the three examined mutants. Lanes are loaded with approximately 0.3 µg protein. (**B**) Protein gel from the alternate purification, showing WT and all mutants. Lanes are loaded with approximately 1 µg protein. (**C**) Western blot for RECQL4 using in-house antibody (described in (1)), showing degradation of R522C, R522H and L638P. Lanes are loaded with approximately 0.1 µg protein.

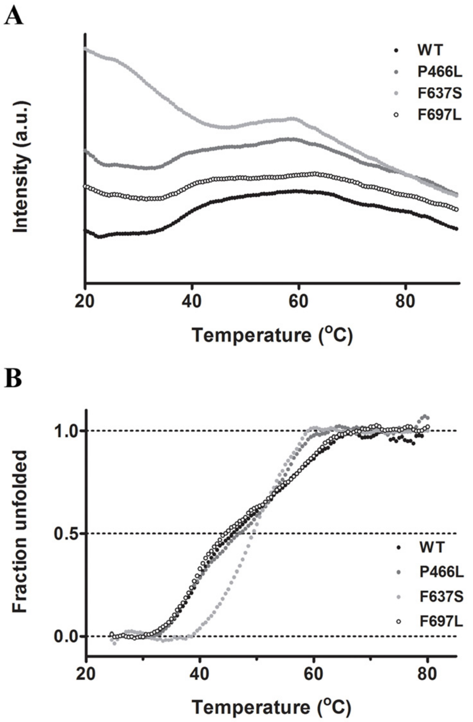

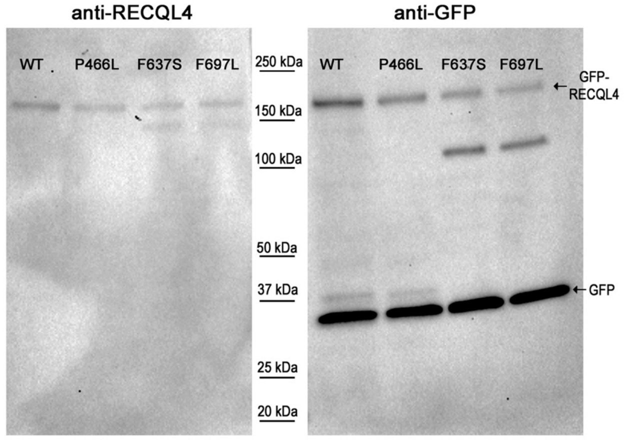

Supplementary Figure 3. Expression of GFP-RECQL4. Lysates from cells used in microscopy experiments (Fig. 6), expressing (WT or mutant) GFP-RECQL4 were examined by Western blotting against RECQL4 (left) and GFP (right). All samples show a band at the expected size of GFP-RECQL4, though free GFP is also present in all samples.

Supplementary Figure 2. Protein unfolding data. (**A**) Raw fluorescence data from the SYPRO® Orange-based thermostability assay, offset for clarity. Protein unfolding manifests as an increase in fluorescence superimposed on the steady decrease inherent to the assay. While F637S shows clean single-step unfolding (a single rise in intensity), both WT and the remaining mutants appear to demonstrate more complex unfolding. (**B**) Melting curves for WT and mutants are generated by quantifying the relative increase in fluorescence upon unfolding, and melting temperatures extracted at the point of 50% unfolding.
